# Bloody tears as initial manifestation of rhino‐orbital mucormycosis

**DOI:** 10.1002/jha2.674

**Published:** 2023-03-13

**Authors:** Xiaoning Wang, Huachao Zhu, Juan Ren, Di Wu, Pengcheng He

**Affiliations:** ^1^ Department of Hematology The First Affiliated Hospital of Xi'an Jiaotong University Xi'an Shaanxi P. R. China

**Keywords:** bloody tears, haploidentical stem cell transplantation, rhino‐orbital mucormycosis

1

A 29‐year‐old male presented with bloody tears in his right eye and right periorbital swelling 2 days later without fever and pain (Figure 1A). He had a history of severe aplastic anemia and underwent haploidentical stem cell transplantation. Next‐generation sequences of blood showed *Lichtheimia corymbifera*. Computer tomography showed opacification of the right sinonasal air spaces (Figure 1B), and nasal endoscopy demonstrated the involvement of the right nasolacrimal duct (Figure 1C). Fluorescence staining of localized debridement of involved tissue culture suggested *Mucor* (Figure 1D), consistent with a diagnosis of rhino‐orbital mucormycosis. Rhino‐orbital mucormycosis is a common clinical form of mucormycosis infection. Sinonasal inoculation is typically the primary site of infection. Mortality associated with invasive mucormycosis is high. Early surgical debridement or excision plays an important adjunctive role.^1^ For this patient, weekly endoscopic clearance of the necrotic material and fungal tissue combined with local and systemic amphotericin B therapy was carried out for 1 month. Finally, the orbital exenteration and disfigurement were avoided, and the right eye was salvaged.

**FIGURE 1:**
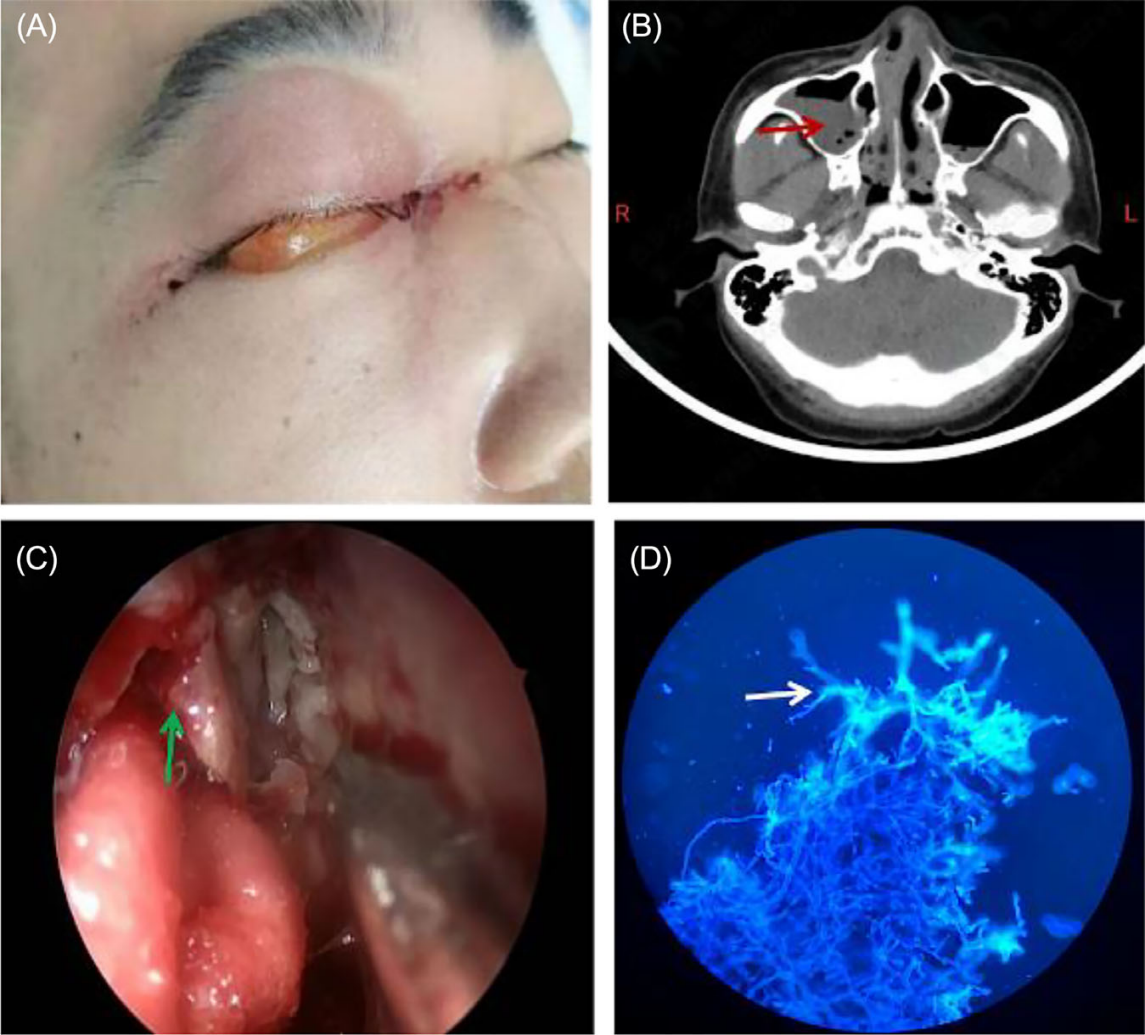
(A) The appearance of the patient right eye 1 day after bloody tears. Visible right periorbital swelling. (B) Computer tomography showed opacification of the right sinonasal air spaces (red arrow). (C) Nasal endoscopy demonstrated the involvement of the right sinonasal and nasolacrimal ducts (green arrow). (D) Fluorescence staining of localized debridement of involved tissue culture suggested *Mucor* (white arrow).

## AUTHOR CONTRIBUTIONS

Xiaoning Wang did the literature search and drafted the manuscript. Huachao Zhu, Juan Ren, and Di Wu provided the photographs of the patient. Pengcheng He reviewed the manuscript. All authors read and approved the final manuscript.

## CONFLICT OF INTEREST STATEMENT

The authors declare no conflict of interest.

## FUNDING INFORMATION

The Key Research and Development Project of Shaanxi Province (2022SF‐13)

## ETHICS STATEMENT

Written informed consent was obtained from the patient for the publication of this case report and the accompanying images, and the case report was approved by the Ethical Committees of the First Affiliated Hospital of Xi'an Jiaotong University.

## CLINICAL TRIAL REGISTRATION

The authors have confirmed clinical trial registration is not needed for this submission.

## PATIENT CONSENT STATEMENT

The authors have confirmed patient consent statement is not needed for this submission.

## Data Availability

Data will be made available on request.
